# Recent Development of Electrospinning for Drug Delivery

**DOI:** 10.3390/pharmaceutics12010005

**Published:** 2019-12-19

**Authors:** Romána Zelkó, Dimitrios A. Lamprou, István Sebe

**Affiliations:** 1 University Pharmacy Department of Pharmacy Administration, Semmelweis University, 7–9 Hőgyes Street, H-1092 Budapest, Hungary; istvan.sebe@gmail.com; 2 School of Pharmacy, Queen’s University Belfast, 97 Lisburn Road, Belfast BT9 7BL, UK

Electrospinning is one of the most widely used techniques for the fabrication of nano/microparticles and nano/microfibers, induced by a high voltage applied to the drug-loaded solution. The modification of environmental conditions, solution properties or operation parameters results in different fiber properties, thus enabling the fine-tuning of functionality-related characteristics of the final product. The latter includes the alteration of the rate and extent of the solubility of drugs, hence the rapid or prolonged onset of absorption.

This Special Issue serves to highlight and capture the contemporary progress of electrospinning techniques, with particular attention to their further pharmaceutical application as conventional and novel drug delivery systems or for tissue regeneration purposes. It comprises a series of 12 research articles and one review, illustrating the versatile researches and teams from 13 different countries, making profound contributions to the field.

Palo et al. investigated a combined technique for the fabrication of bi-layered carriers from a blend of polyvinyl alcohol (PVA) and sodium alginate (SA). The bi-layered carriers were prepared by solvent casting in combination with two surface modification approaches, electrospinning or three-dimensional (3D) printing. An initial inkjet printing trial for the incorporation of bioactive substances for drug delivery purposes was performed. The solvent cast (SC) film served as a robust base layer. The bi-layered carriers with electrospun nanofibers (NFs) as the surface layer showed improved physical durability and decreased adhesiveness compared to the SC film and bi-layered carriers with a patterned three-dimensional (3D) printed layer. The bi-layered carriers presented favorable properties for dermal use with minimal tissue damage. In addition, electrospun NFs on SC films (bi-layered SC/NF carrier) provided the best physical structure for cell adhesion, and proliferation as the highest cell viability was measured compared to the SC film and the carrier with a patterned 3D printed layer (bi-layered SC/3D carrier) [1].

Cherwin et al. developed a novel oxygen therapeutic made from a pectin-based hydrogel microcapsule carrier mimicking red blood cells. The study focused on three main criteria for developing the oxygen therapeutic to mimic red blood cells: Size (5–10 μm), morphology (biconcave shape), and functionality (encapsulation of oxygen carriers; e.g., hemoglobin (Hb)). The hydrogel carriers were generated via the electrospraying of the pectin-based solution into an oligochitosan crosslinking solution using an electrospinning setup. The pectin-based solution was investigated first to develop the simplest possible formulation for electrospray. The production process of the hydrogel microcapsules was also optimized. The microcapsule with the desired morphology and size was successfully prepared under the optimized condition. The encapsulation of Hb into the microcapsule did not adversely affect the microcapsule preparation process, and the encapsulation efficiency remained high (99.99%). The produced hydrogel microcapsule system offers a promising alternative for creating a novel oxygen therapeutic [2].

Unalan et al. prepared antibacterial poly(ε-caprolactone) (PCL)-gelatin (GEL) electrospun nanofiber mats containing clove essential oil (CLV) using glacial acetic acid (GAA) solvent. The addition of CLV increased the fiber diameter from 241 ± 96 nm to 305 ± 82 nm. Along with the increase of the CLV content of nanofibers, the wettability of PCL-GEL nanofiber mats was also increased. Fourier-transform infrared spectroscopy (FTIR) analysis confirmed the presence of CLV, and the actual content of CLV was determined by gas chromatography–mass spectrometry (GC-MS). It was confirmed that the CLV-loaded PCL-GEL nanofiber mats did not have cytotoxic effects on normal human dermal fibroblast (NHDF) cells, while the fibers exhibited antibacterial activity against *Staphylococcus aureus* and *Escherichia coli*. Consequently, PCL-GEL/CLV nanofiber mats can be potential candidates for antibiotic-free wound healing applications [3].

Viet Nguyen et al. developed novel amphiphilic electrospun nanofibers (NFs) loaded with haemanthamine (HAE), phosphatidylcholine (PC), and polyvinylpyrrolidone (PVP), intended for a stabilizing platform of self-assembled liposomes of the active agent. The NFs were fabricated with a solvent-based electrospinning method. The HAE-loaded fibers showed a nanoscale size ranging from 197 nm to 534 nm. The liposomes with a diameter between 63 nm and 401 nm were spontaneously formed as the NFs were exposed to water. HAE dispersed inside liposomes showed a tri-modal dissolution behavior. Amphiphilic NFs loaded with HAE are an alternative approach for the formulation of a liposomal drug delivery system and stabilization of the liposomes of the chemically instable and poorly water-soluble alkaloid [4].

Hakkarainen et al. investigated nozzleless ultrasound-enhanced electrospinning (USES) as a means to generate nanofibrous drug delivery systems (DDSs) for pharmaceutical and biomedical applications. Traditional electrospinning (TES) equipped with a conventional spinneret was used as a reference method. High-molecular polyethylene oxide (PEO) and chitosan were used as carrier polymers and theophylline anhydrate as a water-soluble model drug. The nanofibers were electrospun with the diluted mixture (7:3) of aqueous acetic acid (90% *v/v*) and formic acid solution (90% *v/v*) (with a total solid content of 3% *w/v*). The fiber diameter and morphology of the nanofibrous DDSs were modulated by varying ultrasonic parameters in the USES process (i.e., frequency, pulse repetition frequency, and cycles per pulse). The authors found that the USES technology produced nanofibers with a higher fiber diameter (402 ± 127 nm) than TES (77 ± 21 nm). An increase in burst count in USES increased the fiber diameter (555 ± 265 nm) and the variation in fiber size. The slight-to-moderate changes in a solid state (crystallinity) were detected in comparison to the nanofibers generated by TES and USES. In conclusion, USES provides a promising alternative for aqueous-based fabrication of nanofibrous DDSs for various pharmaceutical and biomedical applications [5].

Paaver et al. investigated and monitored the wetting and dissolution properties of Piroxicam (PRX)-loaded polymeric nanofibers in situ and determined the solid-state of the drug during dissolution. Hydroxypropyl methylcellulose (HPMC) and polydextrose (PD) were used as carrier polymers for electrospinning (ES). The initial-stage dissolution of the nanofibers was monitored in situ with three-dimensional white light microscopic interferometry (SWLI) and high-resolution optical microscopy. They confirmed that PRX recrystallizes in a microcrystalline form immediately after wetting of nanofibers, which could lead to enhanced dissolution of the drug. Initiation of crystal formation was detected by SWLI, indicating that PRX was partially released from the nanofibers, and the solid-state form of PRX changed from amorphous to crystalline. The amount, shape, and size of the PRX crystals depended on the carrier polymer used in the nanofibers and the pH of the dissolution media. The PRX-loaded nanofibers exhibited a quasi-dynamic dissolution via recrystallization. SWLI enabled a rapid, non-contacting, and non-destructive method for in situ monitoring of the early-stage dissolution of nanofibers and regional mapping of crystalline changes during wetting [6].

Preem et al. tested and compared different drug release model systems for electrospun chloramphenicol (CAM)-loaded nanofiber (polycaprolactone (PCL)) and microfiber (PCL in combination with polyethylene oxide) mats with different drug release profiles. The CAM release and its antibacterial effects in disc diffusion assay were assessed by bacterial bioreporters. The release into buffer solution showed larger differences in the drug release rate between differently designed mats compared to the hydrogel release tests. The UV imaging method provided an insight into the interactions with an agarose hydrogel mimicking wound tissue, thus providing information about early drug release from the mat. Bacterial bioreporters showed clear correlations between the drug release into gel and antibacterial activity of the electrospun CAM-loaded mats [7].

Zupančič et al. investigated the effect of electrospinning on the viability of bacteria incorporated into nanofibers. The morphology, zeta potential, hydrophobicity, average cell mass, and growth characteristics of nine different species of *Lactobacillus* and one of *Lactococcus* were characterized. The electrospinning of polymer solutions containing ~10 log colony forming units (CFU)/mL of lactic acid bacteria enabled the successful incorporation of all bacterial species tested, from the smallest (0.74 µm; *Lactococcus lactis*) to the largest (10.82 µm; *Lactobacillus delbrueckii* ssp. bulgaricus), into poly(ethylene oxide) nanofibers with an average diameter of ~100 nm. All of these lactobacilli were viable after incorporation into nanofibers, with 0 to 3 log CFU/mg loss in viability, depending on the species. Viability correlated with the hydrophobicity and extreme length of lactic acid bacteria, whereas a horizontal or vertical electrospinning set-up did not have any role. Therefore, electrospinning represents a promising method for the incorporation of lactic acid bacteria into solid delivery systems, while drying the bacterial dispersion at the same time [8].

Sipos et al. formulated aceclofenac-loaded poly(vinyl-pyrrolidone)-based nanofibers by electrospinning to obtain drug-loaded orally disintegrating webs to enhance the solubility and dissolution rate of the poorly soluble anti-inflammatory active that belongs to the Biopharmaceutical Classification System (BCS) Class-II. Triethanolamine-containing ternary composite of aceclofenac-poly(vinylpyrrolidone) nanofibers was formulated to exert the synergistic effect on the drug-dissolution improvement. The nanofibrous formulations had diameters in the range of a few hundred nanometers. FT-IR spectra and DSC thermograms indicated the amorphization of aceclofenac, which resulted in a rapid release of the active substance. The characteristics of the selected ternary fiber composition (10 mg/g aceclofenac, 1% *w*/*w* triethanolamine, 15% *w*/*w* PVPK90) were found to be suitable for obtaining orally dissolving webs of fast dissolution and potential oral absorption [9].

Vass et al. developed a processable, electrospun formulation of a model biopharmaceutical drug, β-galactosidase. They demonstrated that higher production rates of drug-loaded fibers could be achieved by using high-speed electrospinning compared to traditional electrospinning techniques. An aqueous solution of 7.6 *w*/*w*% polyvinyl alcohol, 0.6 *w*/*w*% polyethylene oxide, 9.9 *w*/*w*% mannitol, and 5.4 *w*/*w*% β-galactosidase was successfully electrospun with a 30 mL/h feeding rate, which is about 30 times higher than the feeding rate usually attained with single-needle electrospinning. According to X-ray diffraction measurements, each component was in an amorphous state in the fibers, except the mannitol, which was crystalline (δ-polymorph). The presence of crystalline mannitol and the low water content enabled appropriate grinding of the fibrous sample without secondary drying. The ground powder was mixed with commonly used tabletting excipients and was successfully directly compressed. β-galactosidase remained stable in the course of the whole processing and after one year of storage at room temperature in the tablets. The results demonstrate that high-speed electrospinning is a viable alternative to traditional biopharmaceutical drying methods, especially for heat-sensitive molecules, and further processing of electrospun fibers to tablets can be successfully achieved [10].

Dwivedi et al. designed a nanocomposite carrier using Poly(d,l-lactide-*co*-glycolide) (PLGA)/gelatin polymer solutions for the simultaneous release of recombinant human epidermal growth factor (rhEGF) and gentamicin sulfate at the wound site to hasten the process of diabetic wound healing and inactivation of bacterial growth. The bacterial inhibition percentage and detailed in vivo biocompatibility for wound healing efficiency was performed on diabetic C57BL6 mice with dorsal wounds. The scaffolds exhibited excellent wound healing and continuous proliferation of cells for 12 days, thus providing a promising means for the rapid healing of diabetic wounds and ulcers [11].

Pisani et al. studied electrospun nanofibers as antibiotic release devices for preventing bacteria biofilm formation after surgical operation. In their work gentamicin sulfate (GS) was loaded into polylactide-*co*-polycaprolactone (PLA-PCL) electrospun nanofibers; quantification and in vitro drug release profiles in static and dynamic conditions were investigated. The kinetics of the GS release from nanofibers was studied using mathematical models. A preliminary microbiological test was carried out towards *Staphylococcus aureus* and *Escherichia coli* bacteria. The prolonged effect of the antibiotic at the site of action can reduce administration frequency and improve patient compliance [12]. 

Pant et al. summarized that electrospinning has emerged as a potential technique for producing nanofibers. The use of electrospun nanofibers in drug delivery has increased rapidly over recent years due to their valuable properties, which include a large surface area, high porosity, small pore size, superior mechanical properties, and ease of surface modification. A drug-loaded nanofiber membrane can be prepared via electrospinning using a model drug and polymer solution; however, the release of the drug from the nanofiber membrane in a safe and controlled way is challenging as a result of the initial burst release. Employing a core-sheath design provides a promising solution for controlling the initial burst release. This paper summarizes the physical phenomena, the effects of various parameters in coaxial electrospinning, and the usefulness of core-sheath nanofibers in drug delivery. It also highlights the future challenges involved in utilizing core-sheath nanofibers for drug delivery applications [13]. 

All the articles presented in this Special Issue represent a small fraction of the great research interest in the field of nanofibrous system applications as drug delivery bases or for tissue engineering purposes. Their diverse and tunable features enable a wide variety of use, which opens a new dimension in the case of their feasible scaling-up.
